# Draft Genome Sequence of Rice Endophyte-Associated Isolate *Kosakonia oryzae* KO348

**DOI:** 10.1128/genomeA.00594-15

**Published:** 2015-06-04

**Authors:** Xianfa Meng, Iris Bertani, Pamela Abbruscato, Pietro Piffanelli, Danilo Licastro, Changhai Wang, Vittorio Venturi

**Affiliations:** a Bacteriology Group, International Centre for Genetic Engineering and Biotechnology (ICGEB), Trieste, Italy; b Jiangsu Provincial Key Laboratory of Marine Biology, College of Resources and Environmental Sciences, Nanjing Agricultural University, Nanjing, China; c Rice Genomics Unit, Parco Tecnologico Padano, Localita Cascina Codazza, Lodi, Italy; d Cluster in Biomedicine (CBM) SCRL–Genomics, Area Science Park, Basovizza, Trieste, Italy

## Abstract

*Kosakonia oryzae* KO348 is an endophytic and plant growth-promoting strain isolated from the roots of rice in Italy. Here, we report the draft genome sequence of *Kosakonia oryzae* KO348.

## GENOME ANNOUNCEMENT

Created in 1960, the genus *Enterobacter* is one of the largest genera within the family *Enterobacteriaceae* (1). Recently, taxonomic studies have resulted in rearrangement of the *Enterobacter* genera (2). As a result, a new genus designated *Kosakonia*, which includes the species *Kosakonia cowanii*, *Kosakonia radicincitans*, *Kosakonia oryzae*, and *Kosakonia arachidis* (2), was proposed, and in 2014, the nitrogen-fixing bacterium *Enterobacter sacchari* was reclassified as *Kosakonia sacchari* (3). *Kosakonia oryzae* has been associated with rice and has been shown to possess plant growth-promoting properties, including the ability to fix atmospheric nitrogen (4).

*Kosakonia oryzae* KO348 was isolated from surface-sterilized roots of the Italian rice cultivar Vialone Nano. Phylogenetic analyses using 16s rRNA and multilocus sequence analysis based on *gyrB, rpoB, infB*, and *atpD* indicate that this novel isolate belongs to genus *Kosakoni*, and here we announce its draft genome sequence. Genomic DNA of *K. oryzae* KO348 was purified following the protocol of the Nextera XT DNA library preparation kit (Illumina) and then used to prepare a sequencing-ready library. Sequencing was performed on Illumina MiSeq platform using 150-bp paired-end reads. Total number of pairs of reads was 5,010,628, representing approximately 10-fold coverage of the genome. We performed the *de novo* assembly using SPAdes v3.0 (5), generating 68 contigs with a maximum length of 462 kbp. The total length of the contig assembly was 5.0 Mbp, and the *N*_50_ length was 172 kbp, assuming a genome size of 5.0 Mbp. The G+C content was 53.9%, similar to that of other *Kosakonia* sequenced genomes. Automated annotation of the *K. oryzae* KO348 draft genome sequence was performed using RAST (6) assigning a total of 4,763 candidate protein-coding genes with 1,045 (21.94%) annotated as hypothetical proteins. A total of 86 RNA coding sequences were also identified. The *K. oryzae* KO348 genome presents several genes related to plant colonization and plant growth promotion, including genes for exopolysaccharide production, flagellar motility, siderophore production, auxin biosynthesis, and the *nif* gene cluster. Furthermore, it contains genes involved in the biosynthesis of type II, IV, V, VI, VII, and VIII secretion systems.

### Nucleotide sequence accession numbers. 

This whole-genome shotgun project has been deposited at DDBJ/EMBL/GenBank under the accession no. JZLI00000000. The version described in this paper is version JZLI01000000.
